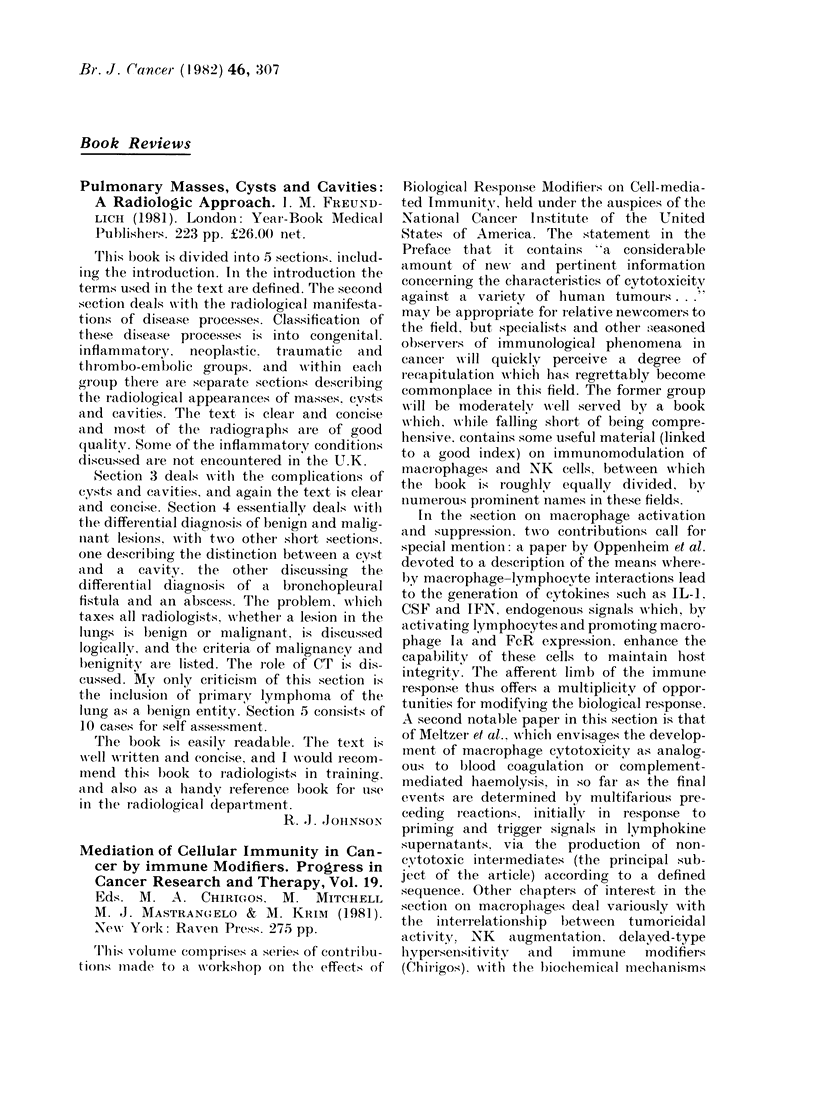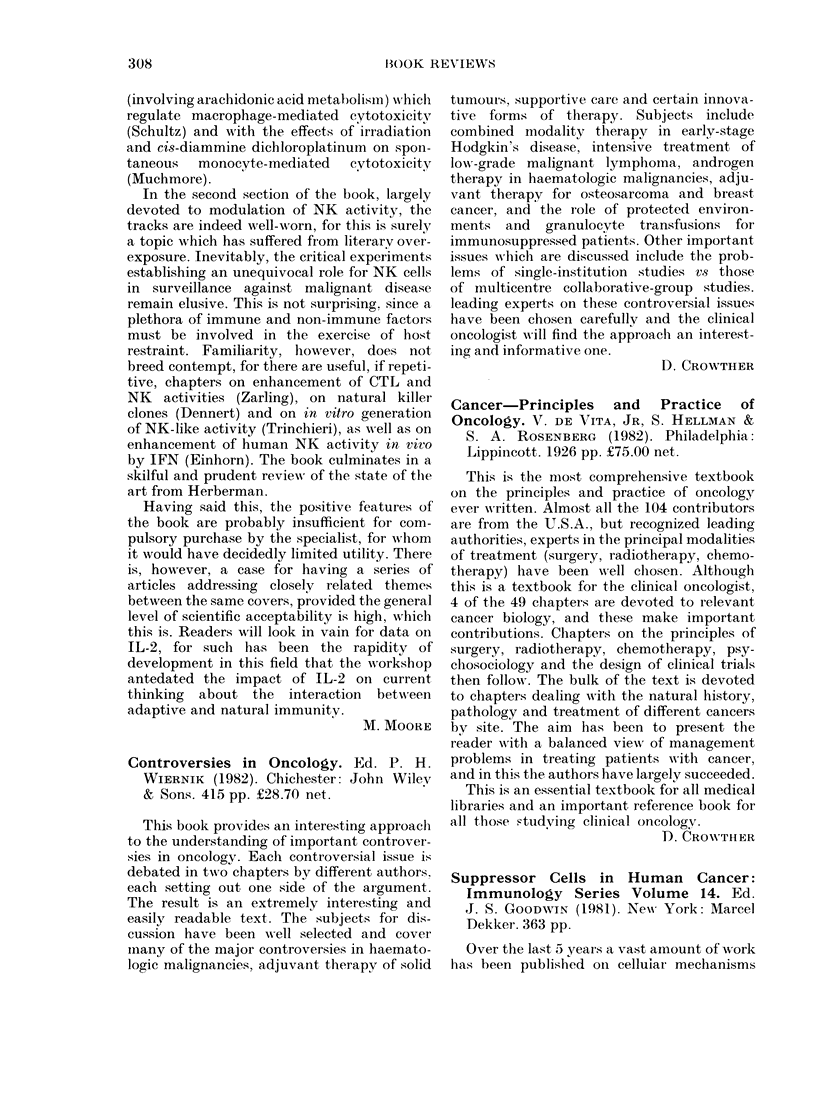# Mediation of Cellular Immunity in Cancer by immune Modifiers. Progress in Cancer Research and Therapy, Vol. 19

**Published:** 1982-08

**Authors:** M. Moore


**Mediation of Cellular Immunity in Cancer by immune Modifiers. Progress in Cancer Research and Therapy, Vol. 19.** Eds. M. A. CHIRIGOS, M. MITCHELL M. J. MASTRANGELO & M. KRIM (1981). New York: Raven Press. 275 pp.

This volume comprises a series of contributions made to a workshop on the effects of Biological Response Modifiers on Cell-mediated Immunity, held under the auspices of the National Cancer Institute of the United States of America. The statement in the Preface that it contains "a considerable amount of new and pertinent information concerning the characteristics of cytotoxicity against a variety of human tumours . . ." may be appropriate for relative newcomers to the field, but specialists and other seasoned observers of immunological phenomena in cancer will quickly perceive a degree of recapitulation which has regrettably become commonplace in this field. The former group will be moderately well served by a book which, while falling short of being comprehensive, contains some useful material (linked to a good index) on immunomodulation of macrophages and NK cells, between which the book is roughly equally divided, by numerous prominent names in these fields.

In the section on macrophage activation and suppression, two contributions call for special mention: a paper by Oppenheim *et al.* devoted to a description of the means whereby macrophage–lymphocyte interactions lead to the generation of cytokines such as IL-1, CSF and IFN, endogenous signals which, by activating lymphocytes and promoting macrophage Ia and FcR expression, enhance the capability of these cells to maintain host integrity. The afferent limb of the immune response thus offers a multiplicity of opportunities for modifying the biological response. A second notable paper in this section is that of Meltzer *et al.*, which envisages the development of macrophage cytotoxicity as analogous to blood coagulation or complement-mediated haemolysis, in so far as the final events are determined by multifarious preceding reactions, initially in response to priming and trigger signals in lymphokine supernatants, via the production of non-cytotoxic intermediates (the principal subject of the article) according to a defined sequence. Other chapters of interest in the section on macrophages deal variously with the interrelationship between tumoricidal activity, NK augmentation, delayed-type hypersensitivity and immune modifiers (Chirigos), with the biochemical mechanisms

(involving arachidonic acid metabolism) which regulate macrophage-mediated cytotoxicity (Schultz) and with the effects of irradiation and *cis*-diammine dichloroplatinum on spontaneous monocyte-mediated cytotoxicity (Muchmore).

In the second section of the book, largely devoted to modulation of NK activity, the tracks are indeed well-worn, for this is surely a topic which has suffered from literary over-exposure. Inevitably, the critical experiments establishing an unequivocal role for NK cells in surveillance against malignant disease remain elusive. This is not surprising, since a plethora of immune and non-immune factors must be involved in the exercise of host restraint. Familiarity, however, does not breed contempt, for there are useful, if repetitive, chapters on enhancement of CTL and NK activities (Zarling), on natural killer clones (Dennert) and on *in vitro* generation of NK-like activity (Trinchieri), as well as on enhancement of human NK activity *in vivo* by IFN (Einhorn). The book culminates in a skilful and prudent review of the state of the art from Herberman.

Having said this, the positive features of the book are probably insufficient for compulsory purchase by the specialist, for whom it would have decidedly limited utility. There is, however, a case for having a series of articles addressing closely related themes between the same covers, provided the general level of scientific acceptability is high, which this is. Readers will look in vain for data on IL-2, for such has been the rapidity of development in this field that the workshop antedated the impact of IL-2 on current thinking about the interaction between adaptive and natural immunity.

M. MOORE